# Complete Genome Sequences of Sulfurospirillum Strains UCH001 and UCH003 Isolated from Groundwater in Japan

**DOI:** 10.1128/genomeA.00236-15

**Published:** 2015-03-26

**Authors:** Takamasa Miura, Yoshihito Uchino, Keiko Tsuchikane, Yoshiyuki Ohtsubo, Shoko Ohji, Akira Hosoyama, Masako Ito, Yoh Takahata, Atsushi Yamazoe, Ken-ichiro Suzuki, Nobuyuki Fujita

**Affiliations:** a Biological Resource Center, National Institute of Technology and Evaluation, Shibuya-ku, Tokyo, Japan; b Department of Environmental Life Sciences, Graduate School of Life Sciences, Tohoku University, Sendai, Japan; c Taisei Corporation, Nase, Kanagawa, Japan

## Abstract

Sulfurospirillum strains UCH001 and UCH003 were isolated from anaerobic *cis*-1,2-dichloroethene-dechlorinating microbial consortia derived from groundwater in Japan. Here, we report the complete genome sequences of strains UCH001 and UCH003.

## GENOME ANNOUNCEMENT

Bacteria of the genus Sulfurospirillum are Gram-negative microaerophilic or facultative anaerobic sulfur reducers belonging to the family *Campylobacteraceae* of the class *Epsilonproteobacteria*. Several species have been identified and isolated from various environmental samples including activated sludge, selenium-contaminated freshwater, anoxic freshwater sediment, soil, groundwater, microbial electrosynthesis system, etc., which suggests that they are ubiquitous organisms with metabolic versatility (1–6). The Sulfurospirillum strains UCH001 and UCH003 were newly isolated from anaerobic *cis*-1,2-dichloroethene (*cis*-1,2-DCE)-dechlorinating microbial consortia derived from chloroethenes-contaminated groundwater in Japan (7). The two strains utilized elemental sulfur, thiosulfate, sulfite, dimethyl sulfoxide, trimethylamine *N*-oxide, nitrate, and oxygen (microaerobic) as electron acceptors, but did not utilize sulfate, nitrite, and chlorinated ethenes (e.g., tetrachloroethene, trichloroethene, *cis*-1,2-DCE, and vinyl chloride). Hydrogen, lactate, pyruvate, fumarate, and formate were utilized as electron donors. Fumarate and malate were fermented. The 16S rRNA gene sequence analysis of the strains UCH001 and UCH003 showed 98.3% and 99.5% similarity to that of S. multivorans DSM 12446^T^ and S. cavolei NBRC 109482^T^, respectively.

The genomes of UCH001 and UCH003 were sequenced by using a combined strategy of shotgun sequencing with a 454 GS FLX Titanium (Roche, Basel, Switzerland), paired-end sequencing with an Illumina HiSeq1000 (Illumina, San Diego, CA, USA), and mate-pair sequencing with an Illumina MiSeq (Illumina). The obtained reads were subjected to k-mer-based trimming by ShortReadManger before use for assembling by Newbler version 2.6. The finishing was aided by GenoFinisher and AceFileViewer (8) and accomplished by additional Sanger sequencing of targeted PCR products. The finished sequences were examined for accuracy by the FinishChecker tool of GenoFinisher.

The complete sequence of the chromosome was analyzed using the Microbial Genome Annotation Pipeline (MiGAP; http://www.migap.org/) for predicting protein-coding, tRNA, and rRNA genes. The functional annotations were performed as previously described (9). The genomes of strains UCH001 and UCH003 consist of 2,606,563-bp and 2,698,323-bp circular chromosomes with 37.6% and 43.9% G+C content and 4 and 3 copies of rRNA operons, respectively.

Calculation of average nucleotide identity (ANI) was performed between genome sequences of 6 type strains (S. arcachonense DSM 9755^T^ [JFBL01000000], S. arsenophilum NBRC 109478^T^ [BBQF01000000], S. barnesii DSM 10660^T^ [CP003333], S. cavolei NBRC 109482^T^ [BBQE01000000], S. deleyianum DSM 6946^T^ [CP001816], and S. multivorans DSM 12446^T^ [CP007201]) using the JSpecies program with default settings for ANIb (http://www.imedea.uib.es/jspecies). The ANIb values of strain UCH001 against type strains ranged from 69% (with strain S. arcachonense DSM 9755^T^) to 81.4% (with strain S. arsenophilum NBRC 109478^T^), showing lower values than the 95% to 96% threshold for distinction of bacterial species (10). This result suggests that strain UCH001 is a novel Sulfurospirillum species. On the other hand, the value of the strain UCH003 was 95.3% with S. cavolei NBRC 109482^T^, so this strain was classified into species S. cavolei (5).

### Nucleotide sequence accession numbers.

The nucleotide sequences of Sulfurospirillum sp. UCH001 and S. cavolei UCH003 chromosomes were deposited in the DDBJ/EMBL/GenBank databases under the accession numbers AP014723 and AP014724, respectively.
